# Supplementary material for: Commonalities among dental patient-reported outcomes (dPROs)—A Delphi consensus study
Source: PLoS One. 2022 Jun 22;17(6):e0268750. doi: 10.1371/journal.pone.0268750 (PMC9216565; doi:10.1371/journal.pone.0268750)
Supplement: Attachment — Submitted filename: PONE-D-22-02098-converted1.docx [file pone.0268750.s001.docx]

PLOS ONE

**Commonalities among dental patient-reported outcomes (dPROs) – a Delphi consensus study**

--Manuscript Draft--

# Abstract

### Background

Improvement of patients’ oral health-related quality of life (OHRQoL) is the main goal ~~for~~ of oral health care professionals. ~~While this is an obvious target~~ However, OHRQoL is not a homogenous construct and how to assess it, is challenging because of the large number of currently available instruments. Investigating available instruments and what they have in common would be necessary for consolidation and standardization of these instruments into a smaller set of tools.

~~A Delphi process using a panel of international dental experts aimed In this study, a panel of international ??? (were they selected from different continents like the OHIP-49 data) eleven dental experts aimed A used the Delphi process to investigate the commonalities among 20 generic multi-item dental patient-reported outcome measures (dPROMs).~~

### Method

If the OHRQoL dimensions including *Oral Function, Orofacial Pain, Orofacial Appearance,* and *Psychosocial Impact* are the fundamental building blocks of the dental *patient’s oral health* experience, these dimensions should be measured by generic multi-item dPROMs. In this study, a panel of international ??? (were they selected from different continents like the OHIP-49 data) 11 dental experts ~~aimed A~~ used the Delphi process to investigate the commonalities among 20 generic multi-item dental patient-reported outcome measures (dPROMs).

A Delphi consensus process with 11 dental experts determined how well ??? 20 of these instruments measured the four OHRQoL dimensions (Have you presented in your results what score on a scale of 0 to 10 had different questionnaires for a particular dimension???)

### Results

~~After reaching a consensus in the Delphi process, dental experts assigned all 20 dPROMs to at least one OHRQoL dimension, i.e., all generic multi-item dPROMs measured either~~ *~~Oral Function, Orofacial Pain, Orofacial Appearance,~~* ~~or~~ *~~Psychosocial Impact~~*~~.~~ All 20 dPRO questionnaires assessed at least one OHRQoL dimension while all four OHRQoL dimensions were measured by at least one instrument, i.e., the four OHRQoL dimensions were essential components of the patient’s oral health experience. This shows that the currently available generic multi-item dPROMs have a lot in common. They share *Oral Function, Orofacial Pain, Orofacial Appearance,* and *Psychosocial Impact* as target constructs. Based on these commonalities, it is plausible and desirable to move towards a single four-dimensional metric to assess oral health impact in all clinical, community-based, and research settings. This step is necessary to advance evidence-based dentistry and value-based oral health care.

### ~~Discussion~~

- The result section as per PLOS One guidelines summarizes the most important results and their significance and no Discussion section is needed in the Abstract

Background

Dental patient-reported outcomes (dPROs) represent what is important to dental patients.(1) Therefore, dental patient-reported outcome measures (dPROMs) determine which dental treatments are the most effective in addressing dental patients’ oral health problems ~~best~~. Two systematic reviews identified ~~153~~ 155 multi-item dPROMs that capture oral disease impact.(2 identified 53 and 3, 102 dPROMs???) These dPROMs provide a multitude of opportunities to measure oral disease impact on the quality of life, but they also ~~provide~~ present challenges to dentists and researches such as which instrument to select and how to interpret instrument scores. The dPROMS essentially measure ~~four attributes(2,3), i.e., they have~~ one or more of the four dimensions of OHRQoL: *Oral Function, Orofacial Pain, Orofacial Appearance*, and *Psychosocial Impact*(4) ~~in common~~(commonalities are what the study is exploring?).

A search for commonalities among the currently available dPROMs could ~~This represents a potential to~~ simplify and consolidate the complex array of ~~currently available~~ existing dPROMs ~~After streamlining dPROMs usage~~ into a smaller and better set of instruments to measure the impact of oral diseases. ~~based on the commonalities among them~~, This would also facilitate the determination of effectiveness and efficacy of dental interventions, ~~would greatly improve because redundancies for the performed outcome assessment can be eliminated~~ and comparison of the results of research studies. ~~can be expanded.~~ Consequently, evidence-based dental practice (EBDP) and value-based oral health care (VBOHC) would greatly benefit from ~~a~~ standardization of patient-reported outcomes assessment.

~~Due to the importance of these commonalities among the current dPROMs for EBDP and VBOHC,~~ (Please avoid unnecessary repetition) ~~the use of study authors’ expert opinion to assign the names of the 153 dPROMs to the four OHRQoL dimensions can only be the first step to investigate the commonalties among these instruments.(2,3).~~ (The non-structured process that these authors performed can be improved quantitatively and qualitatively. First, more dental experts can be involved, allowing for better generalizability of findings. Second, and more importantly, a structured process to determine the link between dPROMs and dimensions would provide substantially more confidence in the results (Is it a critique of the study 2 or 3? Is it required here??? As the aim of those studies was not to investigate the commonalities among the questionnaires). A Delphi technique is such a structured process to derive a consensus about a topic such as the relationship between dPROMs and OHRQoL dimensions. The conceptual advantages of a Delphi process are accompanied by technical advantages of involving many dental experts with international representation and reasonable burden??? using electronic communication (may be placed in Discussion section). This study implemented the Delphi process using a panel of international dental experts and aimed to investigate the commonalities among 53 generic multi-item dental patient-reported outcome measures (dPROMs) used in 20 questionnaires identified in the systematic review of Mittal et al (2019).(2)

# Methods and material

In ~~this~~ the present study, 20 questionnaires that measured oral disease impact were assigned to the four OHRQoL dimensions--*Oral Function, Orofacial Pain, Orofacial Appearance,* or *Psychosocial Impact--* by dental experts using a Delphi process after undergoing a reliability assessment and reaching consensus. The questionnaires contained 36 ~~unique~~ dPROs ~~These dPROs were~~ measured by 53 dPROMs.(2)

## Dental Experts (can they be considered as study subjects????)

The dental experts, comprising of a ~~convenience sample~~ group of 11 international dentists and known to one of the authors (MTJ), ~~(N=11)~~ ~~participated in~~ were selected as dental experts for the study. ~~Inclusion~~ The selection criteria for dental experts were that they had to be a dentist, had to have practiced dentistry in the past year, and were fluent in the English language. ~~Study participants were known to one of the authors (MTJ) and were recruited by him. Study participants They were considered “dental experts.”~~ Each dental expert received ~~a study~~ an invitation by ~~an~~ email with detailed study instructions. The dental experts were ~~asked~~ requested to familiarize themselves with the contents of 20 questionnaires ~~content~~. They were provided the abstract (abstracts of what???) published by the questionnaire author(s), a brief description ~~from the author on~~ of what the questionnaire intended to measure, the reference of ----what?, as well as the questionnaire items (please rephrase). ~~The oral disease-generic dPROMs were identified as instruments that measured oral disease impact across of a broad range of patients.(2)~~ ~~The 20 questionnaires contained 36 unique dPROs. These dPROs were measured by 53 dPROMs.(2)~~

## The Delphi Process

Calibration of dental experts

~~Dental experts were informed about the four dimensions of OHRQoL~~ *~~Oral Function, Orofacial Pain, Orofacial Appearance~~* ~~and~~ *~~Psychosocial Impact~~*~~.~~ The ~~dimension~~ definitions of the four dimensions of OHRQoL: *Oral Function, Orofacial Pain, Orofacial Appearance* and *Psychosocial Impact* were provided ~~for~~ to all dental experts. ~~Even if dental experts had an intuitive understanding of what these OHRQoL dimensions were,~~ They were asked ~~to read the~~ ~~definition for each dimension~~ to familiarize themselves with their meaning and content, even if ~~dental experts~~ they had an intuitive understanding of what these ~~OHRQoL~~ dimensions were.

These dimensions were identified ~~through a systematic collection~~ from 10,778 OHIP-49 OHIP-49 (49-item Oral Health Impact Profile) data ~~in typical~~ of prosthodontic patients and general population subjects ~~of both genders~~ ??~~covering an age range of~~ aged 40 years or more reported in 35 studies conducted in six countries (5,6,7,8). OHIP-49 data formed aggregate secondary data set used for the factor analyses in the Dimension of Oral Health-Related Quality of Life Project.(5) ~~attempts to identify the dimensional structure of the Oral Health Impact Profile.~~ ~~The first stage of this project will suggest a dimensional structure and the second stage will test this structure. The investigated populations are dental patients, represented by prosthodontic patients, and general population subjects. The purpose of the Dimensions of Oral Health-Related Quality of Life Project is to determine the structural validity of the OHIP. The present study is a secondary data analysis using available international data. Target populations were dental patients, represented by prosthodontics patients, and general population subjects in 35 previously completed studies. the ratio of general population subjects to prosthodontic patients was about 3:2. When the project started in June of 2012, data from existing studies were included when they originated from countries with the following characteristics. i) availability of all questionnaire items from a culture/ language with a 49-item OHIP with published psychometric properties about validity and reliability; (ii) availability of both a random sample of general population subjects and a sample of prosthodontic patients; and (iii) availability of adult subjects covering a large age range (min to max age: 40 years) for both genders./ to investigate the number and nature of OHRQoL dimensions measured by OHIP. In this report, we describe our aggregate data set for the factor analyses in the project, which consists of responses to the 49-item OHIP from general population subjects and prosthodontics patients from 6 countries, including a large age range of adult subjects and both genders. data set combines data from 35 individual studies conducted in Croatia, Germany, Hungary, Japan, Slovenia, and Sweden. The combined data set includes 10778 OHIPs from 9348 individuals (N=6349 general population subjects, N=2999 prosthodontic patients). To elucidate the OHIP latent structure, the aggregated data were split into a Learning Sample (N=5173) for exploratory analyses and a Validation Sample (N=5022) for confirmatory analyses. Additional data (N=583) were assigned to a third data~~ ~~set.Data came from 35 studies originating(5) from six countries and contained approximately 10,000 OHIP data. In hypothesis-generating,(6) hypothesis-confirming,(7) and validation analyses(8) the four dimensions of OHRQoL were identified and later confirmed to be applicable to OHRQoL instruments in general.(9)~~

Assignment of PROM to OHRQoL dimensions using the Delphi technique

The data ~~was~~ were collected using an anonymous electronic survey in the English language. The survey was generated using Qualtrics software. Dental experts received an electronic link to the survey, and provided their anonymous responses ~~were collected~~ online by choosing a 7-digit number that was not to be shared with other dental experts or the study organizer. For different ~~each~~ rounds (better to describe first how many rounds etc.???), this number was used to link ~~participants’~~ responses to the corresponding dental expert. While the number identified a particular dental expert, completion of all ratings was anonymous.

The dental experts were asked to assign four scores, i.e., one for each dimension, to 20 questionnaires. ~~identified in a previous systematic review.(2)~~ ~~Oral Function, Orofacial Pain, Orofacial Appearance, and Psychosocial Impact can serve as a simple and clinically appealing set of oral health-related quality of life (OHRQoL) dimensions and therefore provide an opportunity for simpler, but psychometrically improved OHRQoL measurement in the future.~~ The dental experts were asked to indicate with the four scores as to what extent the specific questionnaire assesses each of the four dimensions. The scores could range from 0 to 10 points, where 0 points meant the questionnaire does not measure the OHRQoL dimension at all and 10 points meant the questionnaire measures the dimension perfectly.

The dental experts were requested to assess the 20 questionnaires one by one. They were asked to assign one score each to the four dimensions to rate the extent to which a particular questionnaire assessed that dimension. The score for each dimension could range from 0 to 10 points, where 0 points meant that the questionnaire ~~does~~ did not measure the OHRQoL dimension at all and 10 points indicated that the questionnaire measured the dimension perfectly.

In subsequent rounds of the Delphi, dental experts did not have access to their own previous ratings but were shown summarized group scores including rating medians and interquartile ranges for all four dimensions. ~~For each questionnaire, rating medians and interquartile for all four dimensions were presented.~~ The dental experts were asked to provide ratings again, but this time based on ~~knowing~~ the knowledge of what the group’s assessment was. The dental experts were informed that group ratings can but do not have to influence their own new assessment. The Delphi process was stopped when a consensus was reached, which ~~is~~ has been defined in the following section labelled as “Consensus – Validity assessment of ~~panel~~ expert panel’s ratings.”

Reliability assessment of dental experts ratings

To find the commonalities among dPROs using dental experts’ opinions, the expert ratings needed to be stable over time, i.e., an expert should have the same opinion when asked again. Therefore, a test-retest assessment was performed by asking dental experts twice within two weeks to assign questionnaires to dimensions without knowledge of previous results. Ratings from the first round of the Delphi process were used as “test” results. Before dental experts were given feedback on the group's assessment and ask to continue with the second round of the Delphi process, another survey round (“retest”) was performed to determine test-retest reliability.

~~From the~~ Out of 11 dental experts, only seven provided retest data. We calculated an intraclass correlation coefficient (ICC) using a one-way ANOVA for each expert and for each dimension, resulting in 28 ICCs from 7 (number of raters) times 4 (number of dimensions). Box plots were used to present central tendency and spread of ICCs overall and per OHRQoL dimension. The median ICCs were interpreted according to Fleiss criteria(~~5~~ 16). An ICC <0.40 ~~is~~ was considered “poor,” 0.40 - 0.75 represents “fair-to-good,” and >0.75 ~~is~~ was seen as “excellent” reliability. Which software you used for analysis???

The internal consistency of the panel expert ratings was also determined. Cronbach’s alpha, the overall and by dimension, was computed for the dental expert responses when they were asked which dimension a dPROM belonged to. A Cronbach’s alpha value of 0.90 or higher was adopted as the threshold for sufficient panel expert ratings’ internal consistency.(11)

~~produced similar assessments.~~

~~in the final round overall and by dimension.~~

~~Cronbach’s alpha “measures whether several items that propose to measure the same general construct produce similar scores.”(10) In the case of this Delphi study, Cronbach’s alpha helped to assessed whether dental experts who are asked to assign to which dimension a dPROM belongs produced similar assessments.~~

~~In general, when Cronbach’s alpha is used for questionnaires, scores are often considered~~

~~reliable for “clinical application” when Cronbach’s alpha is 0.90 or higher.(11) Therefore, this value was adopted as the threshold for sufficient panel expert ratings’ internal consistency.~~

Consensus – Validity assessment of ~~panel~~ expert panel’s ratings

A Delphi study does not lead automatically to a consensus; it simply provides an opportunity to reach a consensus.(12) Only if dental experts agree on the topic, can the result be considered a consensus.(12) Therefore, we examined the “dimensionality” of dental expert ratings.

Dimensionality refers to the number and nature of the attributes reflected in the dental

experts’ responses. If dental expert responses could be considered unidimensional, i.e., they would only measure one attribute, it could be assumed that the dental experts have reached a consensus on the topic of commonalities among multi-item dPROMs that measure disease impact. In the case of dental experts’ response ratings being multi-dimensional, i.e., several attributes would be measured, it could be assumed that the dental experts would not have a homogenous opinion about the topic, indicating that a consensus was not reached.

To determine the dimensionality of dental expert ratings, a parallel analysis was performed suggested by Horn.(13) For 9 expert panelists, this technique calculated the 9 expert’s ratings x 9 expert ratings correlation matrix from the 20 PRO questionnaires x 9 dental experts dataset. This technique created a scree plot.(14) The eigenvalues of the sample correlation matrix were plotted against their position from largest to smallest (1, 2, …, 9). The eigenvalue points were related to a straight line. The parallel analysis also created a simulated dataset with 180 observations randomly sampled from 9 independent normal variates. It calculated a 9 x 9 correlation matrix for the simulated data and extracted the 9 eigenvalues, ordering them from largest to smallest. This step was repeated k times (in our case, k = 1000). ~~From 1000 eigenvalues at position 1, the median was calculated. For position 2, 3 to 9, medians were also calculated.~~ From 1000 eigenvalues at positions 1, 2, …, 9, medians were calculated. The 9 eigenvalue medians were related to a dashed line and overlaid on the scree plot. Finally, the intersection of the solid (actual data) and the dashed lines (simulated data) was the cutoff for determining the number of dimensions present in the data. Panel expert ratings would be considered unidimensional when only one eigenvalue would be above the dashed line of the simulated data.

## Assignment of questionnaires to OHRQoL dimensions

Having reached a consensus, median ratings for the group of raters were calculated for each questionnaire and for all four dimensions. Consequently, each questionnaire received four median ratings ~~per questionnaire~~. For example, the “Psychological Impact of Dental Aesthetics Questionnaire”(15) received 0, 0, 9, and 10 points for median ratings for the dimensions *Oral Function*, *Orofacial Pain*, *Orofacial Appearance*, and *Psychosocial Impact*, respectively.

~~From this example, it becomes clear that situations such as 0, 0, 0, and 10 points, indicating a perfect fit for only one dimension, rarely occurs. It is expected that dPRO questionnaires measure several dimensions.~~ From this example, it became clear that situations such as 0, 0, 0, and 10 points, indicating a perfect fit of a questionnaire to only one dimension, could have rarely occurred. It was expected that dPRO questionnaires would measure several dimensions. Therefore, in addition to considering only median ratings of equal to or larger than 5 on the 0 to 10 numeric rating scale ~~as indicators for the dimensions~~, the highest median rating (in previous example, the 10 points) and any other median ratings within 2 points of the highest median rating (in the previous example, the 9 points) were considered to indicate a perfect fit of a questionnaire to a dimension ~~were considered indicators of the dimension~~. Therefore, the example of the questionnaire “Psychological Impact of Dental Aesthetics Questionnaire” measured Psychosocial Impact (score of 10) and *Orofacial Appearance* (score of 9) but did not measure the *Oral Function* (score of 0), or the *Orofacial Pain* (score of 0).

## Two hypotheses about the presence of commonalities among dPROMs

~~To interpret the pattern of dimension indicators~~ To know whether the 20 questionnaires have something in common, two hypotheses were generated:

1. *All 20 dPRO questionnaires assess at least one OHRQoL dimension.*

~~That the dPRO questionnaires have four, and not a smaller number of dimensions in common????, was expressed in another hypothesis:~~

2. *All four OHRQoL dimensions are measured by at least one questionnaire.*

Confirmation of these two hypotheses would be considered evidence ~~that the OHRQoL dimensions~~ *~~Oral Function, Orofacial Pain, Orofacial Appearance~~* ~~and~~ *~~Psychosocial Impact~~* ~~are~~ for the commonalities among generic multi-item dPROMs that measure oral disease impact.

# Results

## Reliability of dental expert ratings

Overall, temporal stability of the questionnaire to OHRQoL dimension assignment was “excellent” according to Fleiss’ guidelines.(16) This was indicated by a median reliability coefficient of 0.86 for all dimensions and all dental experts combined. For three of the four dimensions, the median reliability coefficient exceeded the 0.75 threshold, considered “excellent” reliability, indicative of temporal stability. For the fourth dimension *Orofacial Pain* the coefficient was 0.74 (Figure 1).

Figure 1: Reliability coefficients (N=28) examining the ~~panel~~ expert panel’s ratings~~’~~ temporal stability for the four OHRQoL dimensions.

The dispersion of the reliability coefficients varied across the four dimensions. More variable responses were observed for the dimension *Psychosocial Impact* compared to the other dimensions: *Oral Function*, *Orofacial Pain*, and *Orofacial Appearance*. One ICC estimate (0.20) was an outlier compared to 27 estimates that ranged from 0.56 to 0.99. A Cronbach’s alpha of

0.95 was calculated for all the dental experts’ ratings, and an estimate of 0.90 for all the dimensions. These findings indicated internal consistency sufficient for “clinical application.”(11)

## Validity of dental expert ratings

For all dimensions combined and for each of the four dimensions separately, a dominant general factor??? underlying the dental expert ratings was present (Figure 2), indicating

unidimensionality of dental expert ratings. The differences among dimensions were small. The four plots showed only one eigenvalue exceeded the dashed line of the random eigenvalues, thus illustrating the unidimensionality of each dimension. The validity assessment findings indicated that the dental experts reached a consensus on the topic.

Figure 2: Horn’s parallel analysis to determine expert panelists’ responses dimensionality for the four OHRQoL dimensions. ~~In each of the four plots, only one eigenvalue exceeded the dashed line of the random eigenvalues, thus confirming the unidimensionality of dimension ratings.~~

## Questionnaires to OHRQoL dimensions assignment

*Hypothesis 1 - All 20 dPRO questionnaires assess at least one OHRQoL dimension*

Seven of the 20 dROMs measured a single dimension, seven measured two dimensions, five measure three dimensions, and one instrument measured all four dimensions, confirming the hypothesis (Table 1).

*Hypothesis 2 - All four OHRQoL dimensions are measured by at least one questionnaire.*

*Oral Function, Orofacial Pain, Orofacial Appearance,* and *Psychosocial Impact* were measured by 14, 9, 4, and 13 questionnaires, respectively, confirming the hypothesis.

Confirmation of these two hypotheses was considered evidence for the existence of commonalities among the 20 questionnaires for adult dental patients (Table 1).

Table 1: dPRO questionnaires measuring OHRQoL dimensions. ”x” indicates a median expert rating ≥5 points and ≤2 points within the highest dimension rating. Empty cells indicate median expert ratings >2 points lower than the highest dimension rating. Pl. change ref. No. if required

| **No.** | **Questionnaire** | **Abridged name** | **Authors** | **Publication year** | **Oral Function** | **Orofacial Pain** | **Orofacia l Appea-**  **rance** | **Psycho- social**  **Impact** |
| --- | --- | --- | --- | --- | --- | --- | --- | --- |
| **1** | Rand Dental Health Index(17) | Rand DHI | Gooch, Dolan, Bourque | 1989 |  | x | X | x |
| **2** | Geriatric Oral Health Assessment  Index(18) | GOHAI | Atchison, Dolan | 1990 | x | x |  | x |
| **3** | Jaw Disability  Checklist(19) | JDC | Dworkin,  LeResche | 1992 | x |  |  |  |
| **4** | Dental Impact  Profile(20) | DIP | Strauss,  Hunt | 1993 | x |  |  | x |
| **5** | Mandibular Function Impairment  Questionnaire( 21) | MFIQ | Stegenga, de Bont, de Leeuw, Boering | 1993 | x |  |  |  |
| **6** | Oral Health Impact Profile(22) | OHIP | Slade, Spencer | 1994 | x | x |  | x |
| **7** | Subjective Oral Health Status  Indicators(23) | SOHSI | Locker, Miller | 1994 | x | x |  | x |
| **8** | Dental Impacts on Daily  Living(24) | DIDL | Leao, Sheiham | 1996 | x | x | x | x |
| **9** | Oral Health Quality of Life Inventory(25) | OH-QoL | Cornell, Saunders, Paunovich, Frisch | 1997 | x |  |  | x |
| **10** | Oral Impacts on Daily Performance(2  6) | OIDP | Adulyanon, Sheiham | 1997 | x |  |  | x |
| **11** | Oral Health | OHQoL- | McGrath, | 2001 | x |  |  | x |

|  | Related  Quality of Life- UK(27) | UK | Bedi |  |  |  |  |  |
| --- | --- | --- | --- | --- | --- | --- | --- | --- |
| **12** | Manchester Orofacial Pain Disability Scale(28) | MOPDS | Aggarwal, Lunt, Zakrzewska  ,  Macfarlane  ,  Macfarlane | 2005 | x | x |  | x |
| **13** | Psychological Impact of Dental Aesthetics Questionnaire(  15) | PIDAQ | Klages, Claus, Wehrbein, Zentner | 2006 |  |  | x | x |
| **14** | Jaw Functional Limitation  Scale(29) | JFLS | Ohrbach, Larsson,  List | 2008 | x |  |  |  |
| **15** | Chewing Function Questionnaire  – Alternative  Version(30) | Alt-CFQ | Baba, John, Inukai, Aridome, Igarahsi | 2009 | x |  |  |  |
| **16** | Modified Symptom Severity  Index(31) | Mod-SSI | Nixdorf, John, Wall, Fricton,  Schiffman | 2010 |  | x |  |  |
| **17** | Orofacial Esthetic Scale(32) | OES | Larsson, John, Nilner, Bondemark  , List | 2010 |  |  | x |  |
| **18** | Brief Pain Inventory- Facial(33) | BPI- F | Lee, Chen, Urban, Hojat, Church,  Xie, Farrar | 2010 |  | x |  | x |
| **19** | New Chewing Function Questionnaire(  34) | New-CFQ | Peršić, Palac, Bunjevac,  Čelebić | 2013 | x |  |  |  |
| **20** | Craniofacial | CF-PDI | La Touche, | 2014 |  | x |  | x |

|  | Pain and Disability Inventory(35) |  | Pardo- Montero (not the first author in references), Gil- Martínez, Paris- Alemany, Angulo- Diaz- Parreño, Suarez- Falcon, Lara-Lara,  Fernandez- Carnero |  |  |  |  |  |
| --- | --- | --- | --- | --- | --- | --- | --- | --- |

# Discussion

An international panel of dental experts provided reliable and valid ratings so that a consensus could be reached in a Delphi study regarding how well 20 dental patient-reported outcome (dPRO) questionnaires fit the four dimensions of oral health-related quality of life (OHRQoL).

Each questionnaire assessed at least one OHRQoL dimension, and each of the four dimensions was at least assessed by one questionnaire. Based on these results, it was concluded that multi- item dPROMs have the four OHRQoL dimensions – (em dash) *Oral Function*, *Orofacial Pain*, *Orofacial Appearance*, and *Psychosocial Impact--* in common.

## Comparison with literature

The commonalities among multi-item dPROMs assessing disease impact are fundamental for the meaning and scoring of questionnaires used to measure perceived oral health status because they represent the major attributes measured by these instruments. While dimensionality for individual dPROMs has been studied, e.g., the Oral Esthetics Scale (OES)(36), the dimensionality of larger groups of dPROMs have not been investigated. Therefore, a direct comparison with similar studies is currently not possible.

However, previous studies which assigned names of dPROMs to the four OHRQoL dimensions are informative for interpretation of the current findings. A previous exploratory study, which investigated multi-item generic dPROMs, generated the research hypothesis for the present study.(2) In that study, the names for 53 generic dPROMs could all be assigned to the four OHRQoL dimensions. A second study which used the same methodology of relating dPROM names to the four dimensions through use of a different set of questionnaires yielded identical results.(3) In this study, all specific dPROMs were collected, i.e., instruments designed to measure impact for a specific oral disease and all ~~(N=106)~~ 102 disease-specific dPROMs could also be linked to the OHRQoL dimensions. A study investigating PROMs for pediatric dental patients came to similar conclusions.(~~3~~ may be38) In this systematic review, authors concluded the twelve pediatric dPROMs assessed included items that could be mapped to the four OHRQoL dimensions.(3)

From these studies it can be concluded that, ~~while~~ although ~~oral disease~~-specific and ~~oral health-~~generic dPROMs may target a different set of oral diseases in ~~and while~~ different dental patient populations like adult and pediatric patients ~~dental patient populations have~~ having their specific characteristics, there is no difference ~~of~~ in what they have in common as measurement targets – the four OHRQoL dimensions.

Because dPROMs ~~measuring oral disease impact capture~~ measure what matters to dental patients, their common measurement targets should be related to the reasons why patients seek treatment for their oral health problems. An international study conducted in all six World Health Organization health regions corroborated this.(38) This study found that *Oral Function, Orofacial Pain, Orofacial Appearance*, and *Psychosocial Impact* capture dental patients’ perceived impact of oral health problems worldwide, regardless of whether the patient currently suffers from oral diseases or intends to prevent them in the future.(2) ~~While these findings could be applied to all dental patients, a subsequent analysis restricted to pediatric dental patients confirmed these results.(38)~~

Although OHRQoL is only one among many concepts in the larger group of dPROs, it ~~However, among the dPROs that measure disease impact, OHRQoL~~ is the most important and the most widely used concept worldwide.(1,4) Therefore, comparing our dimensionality findings for dPROMs ~~in general~~ with ~~dimensionality findings~~ those of OHRQoL instrument ~~for the~~ ~~used most widely worldwide, used OHRQoL instrument~~ could provide a deeper insight into dPRO dimensions.

~~Globally, the OHIP is the most widely applied OHRQoL instrument.~~(4) Being the most extensive and a special case of a dPROM, ~~As the most extensive~~ the OHRQoL instrument ~~and being a special case of a dPROM, therefore it~~ should measure the same four OHIP’s dimensions-- ~~Indeed, OHIP’s four dimensions~~ *Oral Function*, *Orofacial Pain*, *Orofacial Appearance*, and *Psychosocial Impact—which have been agreed upon by studies using different* ~~were studied with several~~ methodological approaches ~~and all agreed on OHIP’s four dimensions~~.(39)

## Strengths and limitations

Methodological pros and cons of this study are centered around the basic components of the Delphi study – the expert panel, the investigated dPROMs, and methods used to achieve and demonstrate consensus. ~~There are strengths and limitations of the dental experts, instruments, and consensus.~~

~~Dental experts were the target population of the study.~~ ~~Therefore, selection of a large probability sample from all dental experts worldwide would be the best.~~ In the present study, a small ~~convenience sample~~ group of 11 dental experts was selected – an approach typically ~~performed for~~ adopted in Delphi studies. The participating dental experts may not represent all dentists worldwide.

~~This study discusses generic dPROMs; however,~~ Although specific dPROMs that deal with the specific aspects of particular oral diseases also exist, this study focused on the generic dPROMs because, conceptually, they are applicable to all oral diseases. ~~Because the disease-specific instruments intend to characterize the very specific impact aspects of from a particular disease, they are conceptually less suited to study the overarching themes underlying all dPROMs.~~ As we studied dPROMs for adults, ~~and, strictly speaking,~~ the results may only apply to this age group. ~~However,~~ Nonetheless, the results may be applicable to older children because ~~we do not see any reason why~~ the dPROMs for these children ~~should be different differ from for~~ measure the same major attributes as determined by adult dPROMs. ~~they measure, especially for older children.~~

To assess whether consensus on what???was achieved – a necessary condition to have interpretable results - we used a method that is often applied to investigate the dimensionality of PROMs.(40 it may not be relevant to the topic???) In analogy to PROM items measures one attribute (????), we assumed that dental experts should also measure one attribute (????). Questionnaire items should be unidimensional and dental experts should be unidimensional as well (????). This study found strong evidence that dental experts ratings on the commonalities of dPROMs were unidimensional, ~~attesting~~ indicating that a consensus among dental experts was indeed achieved and ~~providing the necessary condition in order for~~ that the Delphi study results ~~being~~ are interpretable.

## Conclusion

*Oral Function*, *Orofacial Pain*, *Orofacial Appearance*, and *Psychosocial Impact* were solidly identified as the commonalities among generic dPROMs, supporting them as the building blocks of the dental patient’s oral health experience. These findings pave the way towards a four- dimensional metric to assess oral disease impact ~~that is essential for pragmatic implementation oral disease outcome measurement~~ ~~in order~~ to achieve patient-centered and evidence-based clinical decision-making.

# Acknowledgement

We thank Drs. Akanksha Gupta, Christian Hirsch, Daniel Reissmann, Hina Mittal, Ira Sierwald, Katrin Bekes, Oliver Schierz, Radwah Sobieh, Subha Giri, Thiago Nascimento for their help with the study. We thank Dr. Rener-Sitar for her comments to the manuscript draft.

The research reported in this publication was supported by the National Institute of Dental and Craniofacial Research of the National Institutes of Health, USA, under the Award Numbers R01DE022331 and R01DE028059.

# References (please recheck all: their sequence and relevance)

1. Paulson DR, Pattanaik S, Chanthavisouk P, John MT. Including the patient’s oral health perspective in evidence-based decision-making. The Journal of Health Monitoring [published online ahead of print]. 2021 Jul 9. doi: 10.1007/s00103-021-03375-0
2. Mittal H, John MT, Sekulić S, Theis-Mahon N, Rener-Sitar K. Patient-reported outcome measures for adult dental patients: a systematic review. J Evid Based Dent Pract. 2019;19(1):53–70.
3. Rener-Sitar K, John MT, Truong V, Tambe S, Theis-Mahon N. Nonmalignant oral disease-- specific dental patient-reported outcome measures for adult patients: a systematic review. *J Evid Based Dent Pract*. 2021;21(1):101529. doi:10.1016/j.jebdp.2021.101529
4. John MT, Hujoel P, Miglioretti DL, LeResche L, Koepsell TD, Micheelis W. Dimensions of oral-health-related quality of life. J Dent Res. 2004 Dec;83(12):956–60.
5. John MT, Reißmann DR, Dent M, Feuerstahler L, Waller N, Baba K. Factor analyses of the oral health impact profile – Overview and studied population. J Prosthodont Res. 2014;57(3):1–9.
6. John MT, Reissmann DR, Feuerstahler L, Waller N, Baba K, Larsson P, et al. Exploratory factor analysis of the oral health impact profile. J Oral Rehabil. 2014;41(9):635–43.
7. John MT, Feuerstahler L, Waller N, Baba K, Larsson P, Čelebić A, et al. Confirmatory factor analysis of the oral health impact profile. J Oral Rehabil. 2014;41(9):644–52.
8. John MT, Rener-Sitar K, Baba K, Celebic A, Larsson P, Szabo G, et al. Patterns of impaired oral health-related quality of life dimensions. J Oral Rehabil. 2016 Jul;43(7):519–27.
9. John MT, Reissmann DR, Čelebić A, Baba K, Kende D, Larsson P, et al. Integration of oral health-related quality of life instruments. J Dent. 2016 Oct;53:38–43.
10. ~~Internal consistency. In: Wikipedia. 2021.~~
11. Bland JM, Altman DG. Statistics notes: Cronbach’s alpha. BMJ. 1997 Feb;314(7080):572.
12. Cella D, Yount S, Rothrock N, Gershon R, Cook K, Reeve B, Ader D, Fries JF, Bruce B, Rose M; PROMIS Cooperative Group. The patient-reported outcomes measurement information system (PROMIS): progress of an NIH Roadmap cooperative group during its first two years. Med Care. 2007 May;45(5 Suppl 1):S3-S11. doi: 10.1097/01.mlr.0000258615.42478.55. PMID: 17443116; PMCID: PMC2829758.
13. Horn JL. A rationale and test for the number of factors in factor analysis. Psychometrika. 1965;30(2):179–85.
14. Cattell RB. The scree test for the number of factors. Multivariate Behav Res. 1966;1(2):245–76.
15. Klages U, Claus N, Wehrbein H, Zentner A. Development of a questionnaire for assessment of the psychosocial impact of dental aesthetics in young adults. Eur J Orthod. 2006 Apr;28(2):103–11.
16. Fleiss JL. The Design and Analysis of Clinical Experiments. New York: John Wiley & Sons; 1986.
17. Gooch BF, Dolan TA, Bourque LB. Correlates of self-reported dental health status upon enrollment in the Rand Health Insurance Experiment. J Dent Educ. 1989 Nov;53(11):629– 37.
18. Atchison KA, Dolan TA. Development of the geriatric oral health assessment index. J Dent Educ. 1990 Nov;54(11):680–7.
19. Dworkin SF, LeResche L. Research diagnostic criteria for temporomandibular disorders: review, criteria, examinations and specifications, critique. J Craniomandib Disord. 1992;6(4):301–55.
20. Strauss RP, Hunt RJ. Understanding the value of teeth to older adults: influences on the quality of life. J Am Dent Assoc. 1993 Jan;124(1):105–10.
21. Stegenga B, de Bont LG, de Leeuw R, Boering G. Assessment of mandibular function impairment associated with temporomandibular joint osteoarthrosis and internal derangement. J Orofac Pain. 1993;7(2):183–95.
22. Slade GD, Spencer AJ. Development and evaluation of the oral health impact profile. Community Dent Heal. 1994;11:3–11.
23. Locker D, Miller Y. Evaluation of subjective oral health status indicators. J Public Health Dent. 1994;54(3):167–76.
24. Leao A, Sheiham A. The development of a socio-dental measure of dental impacts on daily living. Community Dent Health. 1996 Mar;13(1):22–6.
25. Cornell JE, Saunders MJ, Paunovich ED, Frisch MB. Oral health quality of life inventory (OH-QoL). In: Gary D Slade, editor. Measuring oral health and quality of life. 1997. p. 136–49.
26. Adulyanon S, Sheiham A, Slade GD. Oral Impacts on Daily Performances. In: Slade GD, editor. Measuring oral health and quality of life. Chapel Hill: University of North Carolina, Department of Dental Ecology; 1997. p. 151–60. (Measuring Oral Health and Quality of Life).
27. McGrath C, Bedi R. An evaluation of a new measure of oral health related quality of life-- OHQoL-UK(W). Community Dent Health. 2001 Sep;18(3):138–43.
28. Aggarwal VR, Lunt M, Zakrzewska JM, Macfarlane GJ, Macfarlane T V. Development and validation of the Manchester orofacial pain disability scale. Community Dent Oral Epidemiol. 2005 Apr;33(2):141–9.
29. Ohrbach R, Larsson P, List T. The jaw functional limitation scale: development, reliability, and validity of 8-item and 20-item versions. J Orofac Pain. 2008;22(3):219–30.
30. Baba K, John MT, Inukai M, Aridome K, Igarahsi Y. Validating an alternate version of the chewing function questionnaire in partially dentate patients. BMC Oral Health. 2009 Mar;9(1):9.
31. Nixdorf DR, John MT, Wall MM, Fricton JR, Schiffman EL. Psychometric properties of the modified Symptom Severity Index (SSI). J Oral Rehabil. 2010 Jan;37(1):11–20.
32. Larsson P, John MT, Nilner K, Bondemark L, List T. Development of an orofacial esthetic scale in prosthodontic patients. Int J Prosthodont. 2010;23(3):249–56.
33. Lee JY, Chen HI, Urban C, Hojat A, Church E, Xie SX, et al. Development of and psychometric testing for the brief pain inventory-facial in patients with facial pain syndromes. J Neurosurg. 2010 Sep;113(3):516–23.
34. Peršić S, Palac A, Bunjevac T, Celebić A. Development of a new chewing function questionnaire for assessment of a self-perceived chewing function. Community Dent Oral Epidemiol. 2013 Dec;41(6):565–73.
35. La Touche R, Pardo-Montero J, Gil-Martínez A, Paris-Alemany A, Angulo-Díaz-Parreño S, Suárez-Falcón JC, et al. Craniofacial pain and disability inventory (CF-PDI): development and psychometric validation of a new questionnaire. Pain Physician. 2014;17(1):95–108.
36. Reissmann DR, John MT, Enstad CJ, Lenton PA, Sierwald I. Measuring patients’ orofacial appearance: validity and reliability of the English-language orofacial esthetic scale. J Am Dent Assoc [Internet]. 2019;150(4):278–86. Available from: https://doi.org/10.1016/j.adaj.2018.11.024
37. Shayestehpour S, Sharma K, Mosor E, Omara M, Ritschl V, Stamm T, et al. Patient- reported outcome measures for pediatric dental patients: a systematic literature review and a mapping exercise. J Evid Based Dent Pract. 2021;
38. Bekes K, John MT, Rener-Sitar K, Al-Harthy MH, Michelotti A, Reissmann DR, et al. Pediatric patients’ reasons for visiting dentists in all WHO regions. Health Qual Life Outcomes. 2021;19(1):1–12.
39. John MT. Foundations of oral health-related quality of life. J Oral Rehabil. 2020;(October 2019):355–9.
40. Fink-Hafner D, Dagen T, Doušak M, Novak M, Hafner-Fink M. Delphi Method: Strengths and Weaknesses. Metod Zv [Internet]. 2019;16(2):1–19. Available from: https:/[/www.researchgate.net/publication/337570516](http://www.researchgate.net/publication/337570516)

Figures 1 and 2	[Click here to access/download;Figure;Fig_Delphi study dPRO 01142022.docx](https://www.editorialmanager.com/pone/download.aspx?id=30482924&guid=3164d353-3d9a-4f00-bed7-76c2a09cb0b2&scheme=1)

.2

.4

.6

Fig 1

Oral Function	Orofacial Pain Orofacial Appearance Psychosocial Impact

.8

1

Fig 2

Oral Function

Orofacial Pain

1

5

Factor

10

1

5

Factor

10

Orofacial Appearance

Psychosocial Impact

1

5

Factor

10

1

5

Factor

10

0 1 2

4

6

8

0 1 2

4

6

8

Eigenvalues

Eigenvalues

0 1 2

4

6

8

0 1 2

4

6

8
