# Supplementary material for: Commonalities among dental patient-reported outcomes (dPROs)—A Delphi consensus study
Source: PLoS One. 2022 Jun 22;17(6):e0268750. doi: 10.1371/journal.pone.0268750 (PMC9216565; doi:10.1371/journal.pone.0268750)
Supplement: Attachment — Submitted filename: Response to Reviewers.docx [file pone.0268750.s002.docx]

Journal Requirements:

When submitting your revision, we need you to address these additional requirements.

1. Please ensure that your manuscript meets PLOS ONE's style requirements, including those for file naming. The PLOS ONE style templates can be found at
   <https://journals.plos.org/plosone/s/file?id=wjVg/PLOSOne_formatting_sample_main_body.pdf> and 
   <https://journals.plos.org/plosone/s/file?id=ba62/PLOSOne_formatting_sample_title_authors_affiliations.pdf>.

***Thank you for providing the links, the manuscript has been changed based on the PLOS ONE guidelines.***

2. Thank you for stating the following in the Acknowledgments Section of your manuscript:
   [We thank Drs. Akanksha Gupta, Christian Hirsch, Daniel Reissmann, Hina Mittal, Ira Sierwald, Katrin Bekes, Oliver Schierz, Radwah Sobieh, Subha Giri, Thiago Nascimento for their help with the study. We thank Dr. Rener-Sitar for her comments to the manuscript draft.
   The research reported in this publication was supported by the National Institute of Dental and Craniofacial Research of the National Institutes of Health, USA, under the Award Numbers R01DE022331 and R01DE028059.]

We note that you have provided funding information that is not currently declared in your Funding Statement. However, funding information should not appear in the Acknowledgments section or other areas of your manuscript. We will only publish funding information present in the Funding Statement section of the online submission form. 
Please remove any funding-related text from the manuscript and let us know how you would like to update your Funding Statement. Currently, your Funding Statement reads as follows: 
 [The research reported in this publication was supported by the National Institute of Dental and Craniofacial Research of the National Institutes of Health, USA, under the Award Numbers R01DE022331 and R01DE028059.]

Please include your amended statements within your cover letter; we will change the online submission form on your behalf.

***Thank you for providing this, the funding and acknowledgements have been added to the cover letter.***

3. Thank you for stating the following in your Competing Interests section:
   [NO authors have competing interests.]

Please complete your Competing Interests on the online submission form to state any Competing Interests. If you have no competing interests, please state "The authors have declared that no competing interests exist.", as detailed online in our guide for authors at <http://journals.plos.org/plosone/s/submit-now> 
 This information should be included in your cover letter; we will change the online submission form on your behalf

***Thank you for providing this link, this has been completed.***

Dear Authors,
After appraising the authors’ manuscript in great detail with the effective assistance of 9 reviewers, it is my pleasure to inform you that their manuscript has been granted “ major corrections" status.
Thus, please address our suggestions and provide the revised version at your earliest convenience for a 2nd round of revisions.
Some additional literature that might support your introduction or discussion sections follows: Br Dent J. 2022 Feb;232(4):192. doi: 10.1038/s41415-022-4005-4; Evid Based Dent. 2016 Dec;17(4):109-110. doi: 10.1038/sj.ebd.6401202; Evid Based Dent. 2021 Dec;22(4):143-145. doi: 10.1038/s41432-021-0216-9; Int J Dent. 2020 Dec 29;2020:6621848. doi: 10.1155/2020/6621848.
Thank you for submitting such outstanding work to PlosOne.
Best Regards,
Academic Editor

***Thank you for this suggestion, the articles were reviewed and a reference incorporated into the manuscript.***

***Dear Reviewers,***

***Thank you for your time invested in this manuscript and your edits. Please see our responses to each comment. Revisions are color coded and highlighted in the updated manuscript.***

In text edits from document titled, “PONE-D-22-02098-converted1”.

Response to the Green text:

“Accepted” in text are shown in font color green throughout the manuscript.

“Rejected” change is shown in font color green with yellow highlights throughout the manuscript.

Response to the Red text:

“Accepted” in text are shown in font color red throughout the manuscript.

“Rejected” change is shown in font color red with yellow highlights throughout the manuscript.

Reviewer #1: This research is very interesting, in that it focuses on the quality of life. To often, we think that elimination of disease is what is important and tend to ignore the other aspects that make up good oral health.

***Thank you for your notes, this is greatly appreciated!***

I just have a few comments in regards to the methodology. Firstly, it says 11 international dentists participated, so were only 11 dentists approached? Or were there more dentists who were approached and only 11 participated, and if so please indicate the response rate?.

***Thank you for the question. I have added more information regarding response rate. Please see page 5, in black text, highlighted in yellow.***

Secondly, it would be helpful if more information about these 11 dentists could be provided, such as their highest level of education, years of work experience ( mean - work experience of study group), full time or part time employed and where. Just to provide much more clarity.

***Thank you for the comment and suggestion. I have added more information regarding the dental experts demographics. Please see page 5, in black text, highlighted in yellow.***

Strengths and limitations need to be explained much more clearly. For instance, the fact that convenience sampling was employed and one of the author recruited the participants, instead of random sampling, there could be biasness in the responses.

***Thank you for this comment and suggestion. I have added more information within the Strengths and limitations section. Please see pages 17-18, in black text, highlighted in yellow.***

Reviewer #2: What was the criteria for selection of the inetrnatinal experts. what was the geographical distrubution of the experts. why is no mention of the details of the selection citeria for the expert no dissussed in the text.

***Thank you for the comment and suggestion. Reviewer #1 had the same suggestion as well! I have added more information regarding the dental experts demographics. Please see page 5, in black text, highlighted in yellow.***

Reviewer #3:

Comment #1

The relevance of the study was not clear.
The authors mentioned as an important factor the Delphi process using a panel of international dental experts, however the nationality of the experts was not mentioned, also the sample size of experts were small.
The methods were not concise.

***Thank you for the comments. The Methods and material section has been updated with more information on the demographics of the dental experts and the clarity of methods in the study. Please see pages 4-5, the changes are in colors other than the black original color of the manuscript.***

Comment #2
The Future directions and the impact of the study outcomes was not clear. Importantly, the potential of this study, regarding the proposition of a new validated, clear and optimized tool for dental patient-reported outcomes was not reported.

***Thank you for the comment and suggestion. I have information in regards to potential of this study. Please see page 17, in the paragraph highlighted in Blue. Information on optimizing tolls for dPROs are reported throughout the Discussion section, please see page 15-18.***

Reviewer #4: The term ‘international data’ has been used for OHIP-49 data (ref.2), as the countries where the 35 studies were conducted represented all continents. Is it the same reason for mentioning the raters as the international dental experts i.e., did they represent all continents??? Even if that is the case, one should be cautious about using the term ‘International’ due to the prevailing diversity in different countries and within a particular country as it is related to the generalizability of the study findings.

***Thank you for the comment and suggestions, other reviewers have similar thoughts. I have added more information regarding response rate. Please see page 5, in black text, highlighted in yellow.***

Abstract: If we say ‘A Delphi consensus process with 11 dental experts determined how well 20 of these instruments measured the four OHRQoL dimensions, we have to present in our results as to what score on a scale of 0 to 10 had different questionnaires for a particular dimension. Actually, the aim of the study had been ‘to investigate the commonalities among 53 generic multi-item dental patient-reported outcome measures (dPROMs) used in 20 questionnaires’.
Background: The number of dPROMS investigated in one of the two systematic reviews must be rechecked. Also, the reference number assigned to various references in the reference list, the relevancy of a reference to a topic in the text, the names of authors given in Table 1 have to be reviewed.

***Thank you for the comment in the unclarity of the hypothesis within the abstract and background section. Please see the new Abstract on page 2.***

It is suggested that the authors should collectively and thoroughly revise the manuscript for clarity, brevity, and proper sequencing of sentences and paragraphs. They are requested not to consider the amendments (in green) proposed in the attached modified version of the manuscript, as final. Some sentences (in red) in the text need to be rephrased or explained further to enhance comprehension of the manuscript.

***Thank you for the comment. The manuscript has been reviewed and edited!***

Reviewer #5: Reviewer: This manuscript can contribute to consolidation and standardization of available instruments patients’ oral health-related quality of life.
Please see below some comments:

1. Background, page 4: In the background all references used are from articles by only a researcher.
It would be important to consider studies by others researchers.

***Thank you for the comment and suggestions. The authors and I have looked at the articles suggested by the Editor and have added a cition from the recommendation to the Introduction section. Please see page 3, in black text, highlighted in dark blue.***

The authors used the acronyms: dPROs; dPROMs; OHRQoL; EBDP; VBOHC

We use acronyms because its use saves space and prevent repetition. But, if the reader is not familiar with the acronym and if a paper contains too many, that can be distracting and confusing in itself. Its use will likely detract from the readability of the paper.
Then, we should be prudent in their use of abbreviations. Avoid acronyms in the unless the acronym is used multiple times in the text.

***Thank you for the comment and suggestions. You’re correct in that too many acronyms have taken away from the significance of the use itself. I’ve taken away the acronyms EBDP, VBOHC, as they are not used a lot through the manuscript. Please see page 3, in black text, highlighted in dark blue.***

Dental patient-reported outcomes (dPROs)
dental patient-reported outcome measures (dPROMs)
Oral Function, Orofacial Pain, Orofacial Appearance, and Psychosocial
Impact (OHRQoL)
evidence-based dental practice (EBDP)
value-based oral health care (VBOHC)

2. Material and Methods, page 5:
This sentence needs a reference: “They were provided the abstract published by the questionnaire author(s), a brief description from the author on what the questionnaire intends to measure, the reference, as well as the questionnaire items.”

***Thank you for the comment and suggestions. Each dPROM had an abstract, these abstracts are listed in Table 1. This goes into more detail in Table 1 starting on page 13. I’ve reworded the sentence for more clarity on what is being referenced. Please see page 4, in black text, highlighted in dark blue.***

Material and Methods, page 5:
In this sentence: “These dimensions were identified through a systematic collection of OHIP-49 data in typical dental patients and general population subjects of both genders covering an age range of 40 years or more.”
This acronym (OHIP) lacks the initial definition.

***Thank you for the comment and suggestions. The acronym signifies the questionnaire in discussion. This is mentioned in the paragraph and a few sentences prior. After discussion with the authors, we decided that the intention of the paragraph itself is clear in discussing the Calibration of dental experts. Thank you for the comment! Please see section page 5, in black text, highlighted in dark blue***

Material and Methods, page 05 and page 06:
Where the data may be found? “Study instructions of dental experts”, the survey and results.

***Thank you for the comment and suggestions, other reviewers have similar thoughts. I have added more information regarding response rate. Please see page 5, in black text, highlighted in yellow.***

3. Results: the authors could bring more graphic elements to illustrate the results.

***Thank you for the suggestion of providing more graphic elements to illustrate the results. The authors have discussed this and feel that Fig 1 and Fig 2 provide a great significance in illustrating the results of this study in combination with Table 1. Thank you again for this suggestion.***

4. Discussion:
According to the authors, direct comparison with similar studies is not possible. But the authors could bring similar studies from other areas of health. Furthermore, it is noted that 15 references are citations of an only researcher.

***Thank you for your comment and suggestion. Throughout the Discussion section, verbiage has been added to clarify that this is only a comparison of literature, to discuss what has been written in the past. It is essential that this is written in the discussion section to build on the development of past research on this topic. Thank you again for your suggestion! Please see the Discussion section on pages 15-18.***

References:

References 10, 12, 20, 24 and 25: Check references. Be consistent with referencing Vancouver style across the document.

Reference 40:
Fink-Hafner D, Dagen T, Doušak M, Novak M, Hafner-Fink M. Delphi Method: Strengths and Weaknesses. Metod Zv [Internet]. 2019;16(2):1–19. Available from:
<https://www.researchgate.net/publication/337570516>

Reference 40: insert link to the original source of the article:
<https://mz.mf.uni-lj.si/article/view/184/287>

Reviewer #6: This manuscript applied the Delphi consensus method to study commonalities among 20 pre-selected patient-reported outcomes in the dental treatment category.
The setup description and data analysis methods are sufficiently presented in the text. The Discussion of the results and the cited literature is satisfactory to the claims of the manuscript.
Nonetheless, the methods and discussion can be improved and some important points need to be clarified. Additionally, while the study satisfies its stated hypothesis, it lacks on the delphi method details and its limitations are not sufficiently presented.

1. Better clarification is needed on why only 20 questioners were chosen from a previous meta-analysis by the same authors, what were the inclusion/exclusion criteria? What constituted "a generic dPROM|"? wouldn't assorted specific dPROMs for the most common dental treatment options be better and offer more power to the sample?

***Thank you for your comment and suggestions! The reseason for the choice for the 20 questionnaires in highlighted in the first paragraph in the Methods section. Please see page 4, in the paragraph start highlighted in red. Within that paragraph, the definition of a “generic dPROM” is defined, please see page 4, in the paragraph start highlighted in red. The intention is seaking generic dPROMs rather than specific dPROMs is because the generic dPROMs can be more broadly applied to a range of patients. This is discussed in the Methods section as well. Thank you for your comment!***

2, as this article builds its sample from a previous meta-analysis, a better discussion should include the methodological issues and difficulties associated specific to metal-analysis of dPROs. There is a recent publication by YUN-CHENLIU et al. on this specific issue which I recommend citing here,.

***Thank you for your comments and suggestions!***

3. The Delphi method limitations and biases need to be discussed in a better, more detailed manner, as one assumes it is the main focus of this paper. additionally, more details (in the methods and discussion) need to address the characteristics of the selected experts and the issues of selection bias and group consensus vs. Individual opinion.

***Thank you for the comment and suggestion on the clarity of the Delphi method that has been used. Throughout the Methods section, verbiage has been changed to clarify this. Please see pages 4-10.***

4. the limitations of dPROMs and how do they compare/complement Disease-oriented outcome measurements deserves to be mentioned in the intro and the in the discussion.

***Thank you for the comment and suggestions. More information on comparisons and complements of the dPROMs have been added to the Strengths and limitations. Please see page 17, section start highlighted in red.***

5. I spotted some typos and grammatical errors in the manuscript (e.g. This study implementeds... in the intro) , please re-review.

***Thank you for this comment! The manuscript has been reviewed for typos and grammatical errors. Thank you!***

Reviewer #7: The authors aimed to " to investigate the commonalities among 20 generic multi-item dental patient-reported outcome measures (dPROMs)" using the Delphi process. However I would like to humbly suggest some editions:

1-There are a few spelling, typos and inaccuracies within the text and the references, I would suggest to review the paper;

***Thank you for this comment! The manuscript has been reviewed for typos and grammatical errors. Thank you!***

2-In the 4th line of background it inform 153 dProms, in methods it's says 53 dProms were evaluated, am not sure if this is correct;

***Thank you for seeing this error. Other reviewers have noticed this as well. Please see this change on page 3, highlighted in teal.***

3-Please describe the dimensions definitions

***Thank you for the comment and suggestion! More detailed information has been added to the paragraph on page 3, starting paragraph is highlighted in teal.***

4- Reference 1 is now published, please update

***Thank you for this comment! This has been changed on page 20, Reference 1.***

5- I'm not sure this statement would be correct "the determination of treatment
efficacy of dental interventions would greatly improve..." the questionnaires are assessing the patients' point of view, not treatment efficacy, as per your first statement "Dental patient-reported outcomes (dPROs) represent what is important to dental patients", these tools will represent what is important to the patient which not necessarily will represent what the patient need and treatment efficacy.

***Thank you for suggesting this change. The verbiage of this sentence has been eited. Please see this change on page 3, highlighted in teal.***

6-The dentists chosen to participate in the study are described as dental experts, it would be nice to have more information about this, are they experts in qualitative studies?

***Thank you for the comment and suggestions, other reviewers have similar thoughts. I have added more information regarding dental experts. Please see page 5, in black text, highlighted in yellow.***

7-Am not sure the conclusion is precise, as stated "Oral Function, Orofacial Pain, Orofacial Appearance, and Psychosocial Impact were solidly identified as the commonalities among generic dPROMs, supporting them as the building blocks of the dental patient’s oral health experience." The four dimensionalities were the only options for the dental experts assign each questionnaire, therefore obviously these commonalities would be identified.

***Thank you for the comment and suggestion. After conversation with the authors, we’ve decided to keep this as the closing paragraph to provide clear intent of this manuscript. Thank you!***

Reviewer #8: The presented manuscript discloses pertinent results for dental research, especially, considering the current relevance of the patient-related outcomes. The paper is properly organized and presented. Some adjustments are suggested, as follows:

1) I believe there was a mistyping error of the word “implemented” in the background section phrase “This study implementeds the Delphi process…” (page 04).

***Thank you for this comment! The manuscript has been reviewed for typos and grammatical errors. Thank you! Please see page 4, highlighted in maroon for this specific comment.***

2) I understand that the “Inclusion criteria were that they had to be a dentist, had to have practiced dentistry in the past year, and were fluent in the English language” (page 04). However, did you consider and/or collect any further information on the dentists’ expertise level? Considering the possible methodological impact of the expertise variation among the participants.

***Thank you for the comment and suggestions, other reviewers have similar thoughts. I have added more information regarding dental experts. Please see page 5, in black text, highlighted in yellow.***

3) The Fleiss reference (page 07) should be checked. I consider it should be number 16 as per the references list: “The median ICCs were interpreted according to Fleiss(5).”

***Thank you for your suggestion. I’ve added to this paragraph two times. Please see page 7, in maroon highlight.***

4) The median ratings used for the “Assignment of questionnaires to OHRQoL Dimensions” (page 09), are based on the rate of all the experts (eleven), or only on the seven ones that provided test/re-test data? Consider clarification, as it seems relevant, especially for the abstract section methods description.

***Thank you for the comment and suggestion, please see the reworded paragraph for clarity on page 9, paragraph start highlighted in maroon.***

5) When mentioning a systematic review on pediatric patients (page 16), consider verifying the reference number (3) and changing it for another (37).

***Thank you for catching this! The reference has been lined up and assigned. Pease see page 16, highlighted in maroon.***

6) Consider checking the phrase: “Because disease-specific instruments intend to characterize the very specific impact aspects of from a particular disease, they are conceptually less suited to study the overarching themes underlying all dPROMs.” (Page 18).

***Thank you for the suggestion in rewording this. Please see the start of the paragraph, highlighted in maroon on page 18.***

Reviewer #9: The manuscript deals with a relevant topic, considered one of the dimensions of the tripod of evidence-based practice, which is the patient's perspective regarding their own oral health. A consistent survey was carried out, identifying and testing the main dimensions in 20 existing instruments. This study integrates a line of research, with other works already published by the same group. The results presented are relevant, they bring a contribution in relation to the formulated research question and the possibility of adopting a single instrument. This would allow better comparability between different studies. On the topic of discussion, the authors point out that one of the weaknesses of the study lies in the intentional sample and criteria for choosing the experts to integrate the Delphi study conducted. I agree and this seems to me to be the main weakness of the study. Even so, I suggest that the authors mention if in the criteria for choosing the specialists they included aspects such as time since graduation and practice of the profession, if they act as specialists, if they are from different specialties, if they exercise the profession inserted in different socio-cultural and economic contexts. . Perhaps these are some issues that could eventually impact the results.

***Thank you for the comment and suggestion. Reviewer #1 had the same suggestion as well! I have added more information regarding the dental experts demographics. Please see page 5, in black text, highlighted in yellow.***
